# The Practice of Character Strengths: Unifying Definitions, Principles, and Exploration of What’s Soaring, Emerging, and Ripe With Potential in Science and in Practice

**DOI:** 10.3389/fpsyg.2020.590220

**Published:** 2021-01-27

**Authors:** Ryan M. Niemiec, Ruth Pearce

**Affiliations:** ^1^VIA Institute on Character, Cincinnati, OH, United States; ^2^Other, Cincinnati, OH, United States

**Keywords:** character strengths, VIA classification, VIA Survey, strengths interventions, strengths-based practitioner, strengths-spotting, signature strengths, mindfulness

## Abstract

What does it mean to be “strengths-based” or to be a “strengths-based practitioner?” These are diffuse areas that are generic and ill-defined. Part of the confusion arises from the customary default of practitioners and leaders across many cultures to label anything positive or complimentary as “strengths-based,” whether that be an approach, a theoretical orientation, an intervention, or a company. Additional muddle is created by many researchers and practitioners not making distinctions between very different categories of “strength” in human beings – strengths of character, of talent/ability, of interest/passion, of skill/competency, to name a few. To add clarity and unification across professions, we offer seven characteristics and a comprehensive definition for a character strengths-based practitioner. We center on the type of strength referred to as character strengths and explore six guiding principles for understanding character strengths (e.g., character is plural; character is being and doing) and their practical corollaries. Reflecting this foundation and based on character strengths research, our longstanding work with strengths, discussions with practitioners across the globe, and a practitioner survey asking about strength practices (*N* = 113), we point out several character strengths practices or approaches we describe as soaring (e.g., explore and encourage signature strengths; practice strengths-spotting), emerging (e.g., the integration of mindfulness and character strengths), or ripe with potential (e.g., phasic strengths; the tempering effect; the towing effect). We use the same framework for describing general research domains. Some areas of research in character strengths are soaring with more than 25 studies (e.g., workplace/organizations), some are emerging with a handful of studies (e.g., health/medicine), and others are ripe with potential that have none or few studies yet opportunity looms large for integrating character science (e.g., peace/conflict studies). Using this framework, we seek to advance the exchange and collaboration between researcher and practitioner, as well as to advance the science and practice of character strengths.

Knowing is not enough; we must apply. Willing is not enough; we must do.

‐ Goethe

## Introduction

Over 700 studies on the *VIA* Classification published in the last 10 years; over 15 million surveys administered (VIA Institute, 2021); steeply increasing annual usage of the *VIA* Survey: all reflect a unique precedence of both scholarship and popularity around advancing the science and practice of character strengths. Despite being a young science, there is substantial scientific grounding for practitioners educating and guiding clients. At the same time, the large number of practitioners across the globe applying character strengths presents an opportunity for researchers to explore gaps in the science and practice and continue to advance the work. This is the quintessential bridge between academia’s ivory tower and the practitioner or consumer on main street; it is the dialogue between science and practice.

Myriad definitions of character strengths exist in the literature (e.g., Peterson et al., 2005) and a minimalist definition from the original *VIA* Classification text states they are the routes to the great virtues (Peterson and Seligman, 2004). A more comprehensive definition that sums up the array of cultural, practical, and scientific approaches states: Character strengths are positive personality traits that reflect our basic identity, produce positive outcomes for ourselves and others, and contribute to the collective good (Niemiec, 2018). Said another way, the *VIA* Classification of character strengths is a consensual nomenclature (Peterson and Seligman, 2004), a “common language” to understand what is best in human beings.

Character strengths have been studied across industries (e.g., business/organizations, education, healthcare), professions (e.g., physicians), application areas (e.g., youth, disability), areas of well-being (e.g., mental health, happiness, positive relationships), valued outcomes (e.g., achievement, stress management), and domains of life (e.g., parenting); see VIA Institute (2021) for summaries of the studies in the science of character. One would be hard-pressed to find an area in psychology that has neither some research on character strengths being discussed nor the strong potential for so doing. In part, the recent theory suggests, character strengths are relevant for the full range of human experiences – positive opportunities, as well as adversities and suffering, and the mundane in-between (Niemiec, 2020). Despite the large volume of studies, there remains far more to discover about the practice of character strengths. We attempt here to highlight what we see as patterns or trends in the practice of character strengths.

As we turn to examine strengths-based practices, we intentionally loosely define *practitioner* as any helping professional, such as a psychologist, counselor, social worker, mentor, coach, manager, supervisor, teacher, physician, nurse, health technician, mediator, or professor. Similarly, we loosely define *client* as any person being helped or supported, such as a patient, counseling client, coaches, student, employee, or the general consumer. In addition, we will use the term “character strengths” to refer specifically to the 24 character strengths of the *VIA* Classification (which is the substantial focus of the scientific literature on strengths), while the term “strengths” will refer to the more generic frame of some kind of positive quality. Some studies do not specify the type of strength being investigated, thus, in those cases that lack clarity, we use the term “strengths.”

## What Really Is a Strengths-Based Practice?

In querying thousands of practitioners in workshops across spheres of application (e.g., workplace, education, coaching, counseling) if they are a strengths-based practitioner or have a strengths-based practice, the majority answer “yes.” Then when asked to share what they mean by “strengths-based,” the range of responses is almost as varied as the number of people asked. Unfortunately, “strengths” and “strengths-based” have become so generic in their use that in many cases they have become lackluster and meaningless. This trend is only increasing. Yet, the value of strengths is significant and warrants clear definitions and characteristics of strengths-based practices.

Integration of strengths into practice has been discussed for more than two decades and spans many fields, such as social work (Saleebey, 1996), counseling (Smith, 2006), psychotherapy (Rashid and Seligman, 2018), mindfulness (Niemiec, 2014), organizations (Cooperrider and Whitney, 2005), project management (Pearce), disability (Niemiec et al., 2017), personal/executive coaching (Foster and Auerbach, 2015), and education (Linkins et al., 2015). There is not one pathway, model, or theoretical orientation for describing a strengths-based approach or one set of applications for a strengths-based practice. These are unique to each practitioner and infused into their existing approach as a helping professional. However, we believe there are unifying and relevant characteristics of strengths-based approaches applicable across professions.

A first step is to offer specificity on the type of strengths (discussed later) being examined (i.e., strengths of talent or intelligence are different from strengths of character in definition, malleability, and scope). Therefore, our focus is on character strengths. We suggest, based on a review of hundreds of studies on character strengths (VIA Institute, 2021), discussions with strengths-based practitioners across the globe and our own practices with character strengths, that a practitioner taking a character strengths-based approach employs the following seven elements:

- *Embodies character strength*: the practitioner serves as a role model for character strengths use thus displays character strengths awareness and use as they interact and practice.
- *Educates on strengths*: the practitioner teaches about strengths, explains rationale and importance, corrects misconceptions (e.g., strengths are Pollyannaish or happiology; strengths involve ignoring weaknesses), and offers pathways forward for character strengths use.
- *Energizes*: uplifts and fuels the person out of autopilot tendencies, entrapped mental and behavioral routines, and strengths blindness (Biswas-Diener et al., 2011) patterns.
- *Empowers*: focuses on character strengths to help people move from what’s wrong to what’s strong *and/or* helps them use what’s strong to overcome what’s wrong.
- *Faces adversity*: acknowledges problems and struggles – and when appropriate for the context/relationship, explores them but does not get lost in them, nor allows the positive to be squashed out.
- *Connects*: a character strengths-based approach engenders connections – helping the person become more connected with others, with the world, and particularly with themselves. This strengths connection naturally extends to the practitioner-client dyad.
- *Cultivates seeds*: a character strengths-based approach offers an orientation of cultivating seeds, not just plucking weeds (the negative). Rather than a prescriptive approach, the descriptive language around character strengths is prioritized to build awareness, to explore, and to help the client grow toward positive action (Niemiec, 2014; Linkins et al., 2015).

We propose that these seven action-focused characteristics are essential for an authentic character strengths-based approach. They are central attributes of a practitioner’s mindset. Other beneficial characteristics could be named – such as being goal-oriented or holistic – however, these may not be aligned with certain professions or theoretical orientations. It’s important to understand that any approach, theoretical orientation, or model can be infused with character strengths, and the preceding characteristics can support that, from solution-focused and executive coaching protocols to cognitive-behavioral and psychodynamic orientations to humanistic and social-emotional learning approaches (Niemiec, 2018). As a single unifying definition for a character strengths-based approach (or a generic “strengths-based approach”), we offer the following:

A character strengths-based approach (or practice) is empowering, energizing, and connecting in which practitioners, in their own uniquely personal way and with their own orientation/approach to helping, embody and exhibit their character strengths as they educate clients on strengths and support clients in cultivating their character strengths for boosting well-being and handling adversity.

## Character Strengths Principles

In order to operationalize this definition and its many elements, we next offer a framework of six core principles for strengths-based practitioners to understand and deepen their work. A related, practical corollary accompanies each principle. These are adapted from Niemiec (2018).

### Character Strengths Are Capacities

Character strengths are viewed as capacities for thinking, feeling, and behaving (Park et al., 2004; Peterson and Seligman, 2004). In practical terms, we can think creatively and fairly and have grateful and prudent thoughts; we can feel love, kindness, hope, and humility in our body; and we can behave in ways that are brave, zestful, honest, and forgiving (Niemiec, 2018).

A corollary to this principle is that character strengths can be developed and improved. New research on personality traits shows that personality is more malleable than originally thought (Blackie et al., 2014; Hudson and Fraley, 2015; Roberts et al., 2017), and that the change is not necessarily slow and gradual, which was another previously held assumption. Personality traits can shift for a number of reasons, including normative changes based on our genetics and predictable changes in social role (e.g., getting married, having a child), as well as nonnormative changes. Nonnormative changes include less common but deliberately chosen changes in one’s social role (e.g., joining the military) and atypical life events (e.g., going through a trauma; Borghans et al., 2008). In a study of the latter, the character strengths of gratitude, hope, kindness, leadership, love, spirituality, and teamwork all increased in a United States sample (but not a European sample) 2 months after the September 11, 2001 attack on the World Trade Center in New York City (Peterson and Seligman, 2003). Ten months later these character strengths were still elevated but to a lesser degree.

Deliberate interventions focused on improving a part of our personality such as our character strengths also affect personality change. Intervention studies show that such intentional changes can have a positive impact (Yeager et al., 2014; Hudson and Fraley, 2015; Roberts et al., 2017). Practitioners can help clients tap into their character strengths capacities.

### Character and Character Strengths Are Dimensional

Character strengths are expressed in degrees – we have degrees of creativity, honesty, zest, and so on. As opposed to a categorical or diagnostic approach where one has a disorder, condition, or not, these strengths are measured and expressed as “continuous traits,” in that any character strength can show up across a wide continuum of more and less (Miller, 2013). For practitioners, it’s important to reflect on dimensionality so that clients are not lost in all-or-none labels and placed in the creativity box or the teamwork box or as being empty in the self-regulation or humility boxes.

A corollary is that character strengths can be overused and underused along a dimension of character strengths expression. Any of the 24 character strengths can, in a given situation, be brought forth “too much” (overuse) or “too little” (underuse) which are viewed as strengths expressions or lack thereof that has a negative impact on oneself or others (Niemiec, 2019a). Too much curiosity is nosiness and too little can be apathetic, while an overplay of prudence is stuffiness and an underuse of it can be reckless.

### Character Is Plural

As Chris Peterson (2006) often explained, the character is plural. This means people are not simply kind or humble, brave or hopeful, or honest. Rather, people display a variation, multiplicity, and uniqueness in their character strengths profile that informs the rich tapestry of an individual’s character.

A practical corollary is that character strengths are not expressed in isolation but in combinations or constellations (Peterson, 2006; Biswas-Diener et al., 2011; Niemiec, 2018). It’s likely that as situations become increasingly complex or challenging, the array of character strengths being expressed increases. For example, a person making a career transition may find themselves leaning strongly on a panoply of character strengths, whereas a person who is doing their standard job on autopilot is likely to be expressing fewer character strengths and with less intensity.

This can also be framed using the relational concept that character strengths are interdependent – they “inter-are” (Niemiec, 2012), to echo the Buddhist concept of interbeing (Nhat Hanh, 1993). The character strengths all relate to one another (McGrath, 2013) to some degree and these interactions might enable or hinder the expression of one another (Peterson and Seligman, 2004).

### All 24 Matter

An important pursuit in the creation of the *VIA* Classification was that whichever character strengths and virtues were included that they be ubiquitous across people, universal to the human experience (Peterson and Seligman, 2004). Research was conducted on these strengths among people in remote cultures (Biswas-Diener, 2006) and surveys across nations (Park et al., 2006; McGrath, 2015) that support this principle. The character strengths, although varying in degrees, are part of being human.

A practical corollary to “all 24 matter” is that the importance of any given strength will vary by the situation or the intended consequence. For example, hope and zest are the character strengths found repeatedly to have the strongest links with happiness (Park et al., 2004; Proctor et al., 2009), with some causal evidence (Proyer et al., 2013b). In terms of a different outcome or consequence, achievement, it’s likely that perseverance is going to matter in a significant way (Lounsbury et al., 2009; Wagner et al., 2019). While all 24 matter, how they matter will vary by person and situation.

### There Are Many Kinds of Strengths

The category of character strengths is not the only type of strength human beings express. Strengths categories can be and should be differentiated. This principle is important for the science of strengths to grow. A number of distinct types of strengths can be identified – talents (abilities or intelligences), skills (competencies), interests (passions), values, and resources.

Talents are hardwired abilities that encompass what we do naturally well; the multiple intelligences of Howard Gardner (1983) represents one set of examples. Skills develop through learning and practice, such as job skill-building with computer programming or presentation skill development or personal skill development around anger management or diversity skills training. The strength category of interests reflects our passions in life, those activities we are drawn to especially during leisure time; such as sport, art, and music. Resources are a strength category that is external to us; examples include having supportive friends, living in a safe neighborhood, and belonging to a spiritual community. Values are what we internally hold dear and reside in our thoughts and feelings; they say nothing about the action we actually take. A value for hard work does not equate to putting that value into action without turning to character strengths of perseverance and zest to transform value into behavior.

A corollary to this principle, we hypothesize, is that character strengths are the central mechanisms that allow these other strength categories to operate. For example, if someone has a talent for playing the guitar, they need to invest in ~10,000 h of deliberate practice over a 10 years period (Ericsson and Ward, 2007) to really develop that talent/intelligence; this requires depths of perseverance, self-regulation, hope, prudence, creativity, and other character strengths to maximize that talent. In this way, character strengths are the driving force for other types of strengths (Niemiec, 2018).

### Character Is Being and Doing

The work of character strengths involves being *and* doing. For “being,” character strengths reflect our identity, self-understanding, and supporting people to be themselves. For “doing,” character strengths are expressed in behaviors/actions. There is support for both approaches in the literature: Research on signature strengths reflects identity – “being” true to one’s best qualities (e.g., Seligman et al., 2005). As researcher Rhett Diessner observed: “Traits are ontologically closer to the core of human being than is thinking or reasoning” (Diessner et al., 2009, p. 255). At the same time, there is an abundance of research linking character strengths and different types of action and outcomes – which can be viewed as our “doing” – putting one’s best qualities into action (e.g., Gander et al., 2013). A practical corollary is a connection with the overarching self-development goals of authenticity and goodness (Niemiec, 2014). Individuals aspiring to be more authentic in life may focus on the “character is being” element (i.e., being authentic), while those striving to do more good in the world may resonate with the “character is doing” element (i.e., doing good).

## The Practice of Character Strengths: Soaring, Emerging, or Ripe with Potential?

To build off the preceding principles and elements and definition of a strengths-based approach, and to further our hypotheses and experiences with character strengths-based practice, we administered a second section, 22 question survey using the Survey Monkey platform. The first section asked participants to rate themselves on each of the criteria of the “Checklist for Strengths-Based Practitioners” in Niemiec (2018; results are discussed in Table 1). The second section of the survey asked a number of questions about character strengths use in practice (results are discussed in Table 2). To recruit participants, we targeted audiences likely to be practitioners familiar with character strengths, including a robust Facebook group dedicated to character strengths knowledge and use, a personal invitation during a large, weekly, international, online community event dedicated to the topic of character strengths, and through the second author’s LinkedIn profile. The survey was open for 2 weeks in May 2020. A total of 113 individuals responded to the first section of the survey and 106 individuals completed both sections. Of the 113 respondents, 62 self-identified as therapists, counselors, or coaches. The remainder represented teachers, managers, and other professionals with some aspect of a helping role.

**Table 1:** Results from section 1 of the practitioner survey (*N* = 113). Each item from the Character Strengths Practitioner checklist in Niemiec (2018) is shown, including those not asked (noted with N/A).

**Average score from 1 (never) to 100 (always)**	**How often do you do the following with clients?**
46	Administer the online *VIA* Survey prior to or at the first meeting with a client.
64	Review the results of the *VIA* Survey and co-explore the connections between the results and the client’s life.
75	Ask several questions that assess and explore what is best in the person.
67	Offer an equal amount of exploratory questions that target strengths compared with problems/weaknesses.
71	Address the various categories of human strengths, in addition to character strengths, such as abilities/talents, skills/competencies, interests/passions, and external resources.
71	Deliberately use character strengths to offer an insight or a reframe on problems, conflicts, and stressors.
60	Label character strengths in the moment during sessions and offer an explanation for the strength you spotted.
46	Offer summary feedback on your client’s character strengths in every meeting.
67	Consciously use your own character strengths, especially your signature strengths, during client meetings.
51	Prepare for meetings by reviewing your client’s signature strengths before you meet with them.
37	Adhere to a structured model to character strengths (e.g., aware-explore-apply) that is embedded in your approach to helping clients.
63	Collaboratively discuss and draw direct links between client goals and their character strengths.
N/A	Really “see” and understand who your clients are – their core identity, by seeing their signature strengths in action.
N/A	Not only know but offer appropriately timed interventions that fit with their personality and issues.
N/A	Reflect on what you did well (including the strengths you used) with a client immediately following the session.

**Table 2:** Frequency of responses to character strengths-based questions (*N* = 106).

Question	Response options	Percentage of respondents (rounded)
How do you describe your character strengths practices? (Example of *formal* is planning out ways to strategically boost particular strengths; example of *informal* is asking questions about strengths as it comes up in the discussion)	Mainly formal	14%
	Mainly informal	40%
	50–50 formal/informal	40%
	Other	6%
How do you use character strengths in your work?	Character strengths are a supplemental tool or technique	37%
	Character strengths are foundational to the way I do my work	34%
	Character strengths are used by me personally to help support my working mindset.	15%
	Character strengths are new to me	8%
	Character strengths are a personal interest only	6%
What are the most important components of a character strengths practice? (choose up to 4)	Taking action with character strengths	58%
	Self-reflection on optimizing signature strengths use	58%
	Self-reflection on character strengths overall	48%
	Informal character strengths-spotting activities in others	44%
	Sharing character strengths with others	38%
	Planning for action with character strengths	29%
	Formal character strengths-spotting activities in others	28%
	Informal character strengths-spotting activities in self	24%
	Feedback from others on character strengths they see (e.g., Character Strengths 360)	20%
	Formal character strengths-spotting activities in self	14%
	Collecting feedback on character strengths actions	8%
How often do you bring character strengths into your practice?	Always (every interaction I have involves character strengths discussions and questions)	12%
	Frequently (most interactions I have involve character strengths discussions and questions)	48%
	Sometimes (some interactions I have involve character strengths discussions and questions)	25%
	Occasionally/Rarely (every once in a while I bring up character strengths)	11%
	Never	2%

The instructions offered to participants were minimal, focusing on the purpose of the survey as an informal gathering of information; and that the intended use of the results was to explore, in aggregate, how character strengths practices are emerging. Participants were not required to provide a name or e-mail although most did. Due to the mostly “character strengths” context mentioned, it is likely that participants were responding to the strengths-oriented questions with a mindset focused on “character strengths,” however, we did not specifically ask participants which type of strengths (e.g., character strengths, talents, skills, interests, etc.) they used in practice nor did we define these terms, therefore we cannot be certain participants were responding to questions with the 24 character strengths of the *VIA* Classification in mind. Our intention with the survey was to gather general impressions of practitioners’ experiences with character strengths and to begin to understand potential trends in the utilization of character strengths-based practices with clients and in personal growth. Table 2 shows the questions we asked in part two (with forced-choice format as noted) and the results in percentages.

The survey results reveal the use of character strengths practices to be relatively high, with 60% describing their use as always or frequently. About one-third (34%) view character strengths as foundational to their strengths-based practice while 37% view character strengths as a supplemental approach or adjunctive technique to their work. A small percentage (14%) of practitioners takes a formal approach in mapping out their strengths interventions with clients. This might reflect how character strengths practices are new and/or amorphous for many practitioners who perhaps do not feel equipped to map out formal structured approaches.

A general impression from these results is that character strengths continue to gain traction yet there is substantial opportunity for expansion and deepening: becoming more knowledgeable about the range of practices, and more routine and nuanced with the work. That said, this survey should not be viewed as a reflection of any field or profession as a whole as it was intentionally targeted narrowly – toward those who identify as engaging in strengths-based practices (and most likely, character strengths-based practices in particular). We imagine a normative survey of a particular practitioner profession would yield lower percentages in terms of character strengths engagement and application.

The following three subsections discuss the practice (“the how”) of character strengths, using a framework of what’s soaring, what’s emerging, and what’s ripe with potential. The purpose of these descriptive labels is to illuminate a range of practices, highlight strong areas, and offer concrete practices for practitioners to consider and for researchers to examine. They are based on an amalgamation of our experiences in practice, educating, and consulting, and conversations with strengths-based practitioners across the globe over a 10-year period, research on strengths practices and character strengths interventions, and the aforementioned survey. Of these, the greatest weight is given to the science of character strengths, followed by our experiences and our discussions with leading practitioners.

*Soaring* refers to practices that are popular and appear to be well-established among practitioners who work with character strengths. These approaches are research-based and/or solidly grounded conceptually. A soaring practice does not mean it is a foregone conclusion that the activity or approach will be successful for clients, nor that there is a mountain of research. In all cases, the science of character strengths is in need of deeper examination of the many nuances, dynamics, and applications. In some cases, soaring practices are those in which the practice of character strengths precedes the development of an extensive science of character strengths.

*Emerging* refers to practices that are increasing in popularity among practitioners familiar with character strengths. In such cases, the science is unfolding and does not reach the soaring point because either the science is too sparse or it’s not a tip-of-the-tongue approach for practitioners.

*Ripe with potential* refers to practices that have substantial promise and could be explored and developed for client benefit. These need scientific investigation. All are practices that strike a chord with practitioners and are being deployed with clients on a case-by-case basis. In some cases, the science might be ahead of the practice in that there is a strong scientific backing for the underlying philosophy/approach outside of the strengths field, however, practitioners are not aware of it or routinely using it.

These three categories – soaring, emerging, and ripe with potential – are not a ranking of priorities in practice nor do they represent a hierarchy of approaches.

## Soaring Practices

### Prioritize Strengths Over Deficits

Due to an entrenched negativity bias coupled with consistent research that bad is stronger than good (Baumeister et al., 2001), it is a paradigm shift for practitioners to teach their clients to look for strengths and to reframe struggles. The degree to which practitioners educate on this – and consistently prioritize strengths – varies significantly but it is becoming more common. Numerous studies have found a strengths-focused approach to be superior to a deficit-focused approach. For example, focusing on strengths prior to student exams boosted optimism and buffered negative emotions, distress, and the decline of well-being compared to focusing on weaknesses (Dolev-Amit et al., 2020). Other studies comparing strengths with weaknesses have revealed benefits for the former group for clinical depression outcomes (Cheavens et al., 2012), for personal growth outcomes (Meyers et al., 2015), and for perceived competence and intrinsic motivation (Hiemstra and Van Yperen, 2015). While this does not imply a unilateral superiority of a strengths-focus, nor is it a rationale to ignore deficits, it clearly encourages and challenges practitioners to question their existing deficit-laden approach.

In our practitioner survey, the majority (84%) of respondents said that they assess and explore what is best in the person at least half the time; only 8% said they rarely or never do this. This leads us to the next soaring practice.

### Use the *VIA* Survey

The *VIA* Survey (also referred to as the *VIA* Inventory of Strengths) is a psychometrically valid tool used to assess the 24 character strengths. It has undergone extensive revisions over the years based on published analyses (McGrath and Wallace, 2019), as well as a technical manual for development and psychometrics on its various versions (McGrath, 2017). Researchers utilize short forms, virtue measures, reverse-scored items, and direct measures of signature strengths (McGrath and Wallace, 2019). Practitioners use the *VIA* Survey to start strengths conversations with clients, to build strengths awareness, to combat strengths blindness, to overcome client preoccupation with weaknesses/flaws, to enrich exploration of problems, and to catalyze interventions that foster client goals.

With over 15 million surveys administered and a steady increase each of the last 5 years, the popularity of the measure is clear. Its use in university positive psychology and well-being courses for students is commonplace and is strongly inclining in organizational/business and educational settings and counseling clinics. In our practitioner survey, practitioners administered the *VIA* Survey to each of their clients by the first meeting less than half the time (see Table 1 for the items and average scores for this practice and for several other practices we assessed using the “Checklist for Strengths-Based Practitioners” in Niemiec, 2018). The number of practitioners who administer the *VIA* Survey in later sessions is unknown.

### Explore and Encourage Signature Strengths

Signature strengths are those character strengths highest in an individual’s *VIA* Survey results and are defined as involving the three E’s – character strengths that are *essential* or best reflect who the person is at their core; *energizing* in that expressing the strength is uplifting and elicits an increase in energy levels; and *effortless* in that the expression is easy and natural (Niemiec and McGrath, 2019).

Despite only having a few sentences in the 800-page *VIA* Classification text that introduced this consensual nomenclature (Peterson and Seligman, 2004), the concept, research, and practice of signature strengths has received substantial attention, especially in the science of positive psychology. A meta-analysis was published on the intervention, use a signature strength in a new way (Schutte and Malouff, 2019), which involves subjects identifying one of their highest strengths from their *VIA* Survey results and then using that signature strength in a new way each day, typically for 1 week. The meta-analysis found that in randomized controlled studies, this intervention boosted happiness, flourishing and strengths use, and decreased depression. The practical way this intervention is framed in studies makes it easy for practitioners to apply it with clients.

In the practitioner survey, 58% said they self-reflect on signature strengths, that they use their own signature strengths during sessions/meetings about 63% of the time, and they prime themselves to their client’s signature strengths before meetings (Fluckiger et al., 2009) ~45% of the time.

### Engage in Strengths-Spotting

Operationalized as the SEA model (Niemiec, 2018), the steps of character strengths-spotting involve the practitioner spotting/labeling the strengths they see in action, explaining with rational/behavioral evidence how they saw the strengths expressed, and offering appreciation – pointing out the perceived value of the strength from a perspective of emotionality, meaning, linkage with goals/outcomes.

The spotting of character strengths in oneself or others is easy to hold as an assumption that it’s useful and practical and neglect its scientific investigation. In addition, many character strengths intervention studies embed strengths-spotting in the intervention in that the subjects identify their top strengths from a list, use their top five strengths on the *VIA* Survey, or consider a strength they value and want to expand upon and thereby the aspect of character strengths-spotting is not examined separately. That said, a couple of recent studies have looked at strengths-spotting itself and found benefits relating to positive affect, classroom engagement, and need satisfaction (Quinlan et al., 2019); and in an analysis of behaviors associated with strengths-spotted (written about), a variety of valued outcomes were found including empathy, spontaneous affection, helpfulness, friendship, letting go, and speaking positively (Haslip et al., 2019).

While practitioners might not use character strengths-spotting in every meeting, we view this as a soaring approach that has taken hold. In many cases, it is the first step practitioners use when sharing about character strengths with clients or encouraging them to take action. More than half (52%) of the practitioners surveyed use at least one type of strengths-spotting intervention with clients.

### Draw the Well-Being/Happiness Link With Character Strengths

One of the character strengths outcomes most investigated has been well-being, in which various measures of flourishing and related concepts such as thriving, life satisfaction, emotional happiness, and elements of flourishing (e.g., positive relationships, accomplishment, meaning) have been positively correlated with character strengths. From early studies (Peterson et al., 2005), to recent studies (Wagner et al., 2019), to cross-cultural work (Shimai et al., 2006), to direct causal work (Proyer et al., 2013a) and multiple intervention studies (e.g., Gander et al., 2013), the alignment of well-being and/or happiness indicators and character strengths is one of the most consistent positive findings in the field of positive psychology.

While broad character strengths work can increase one’s well-being and decrease ill-being, many practitioners narrow in on what some researchers have dubbed “the happiness strengths” (Littman-Ovadia et al., 2016). So-named because of their consistent link with happiness across several studies, cultures, and populations (e.g., Park et al., 2004), the five strengths are zest, hope, love, gratitude, and curiosity. Many practitioners appreciate the single-intervention simplicity and straightforward approach of targeting one of these character strengths in clients. Niemiec (2018) offers evidence-based interventions for each, referred to as activate your zest, best possible self, loving-kindness meditation with strengths, gratitude letter/visit, and boosting curiosity through novelty. Caveats accompany this approach such as that there are many ways to happiness through strengths (not just targeting one or more of these five); that if a client is not high in them it does not mean they cannot boost happiness; and that being high in them is not a happiness guarantee.

## Emerging Practices

### Draw the Adversity/Resilience Link With Character Strengths

While we’d like to say this is soaring in popularity, it is clear practitioners focusing on character strengths in the first couple decades of the *VIA* Classification have veered toward well-being, sometimes exclusively when discussing strengths. Theories have been developed that character strengths are at the core of both positivity/opportunity and adversity/suffering. Numerous character strengths functions on the adversity/suffering side include the buffering, reappraisal, and resilience functions (Niemiec, 2020). There are studies looking at character strengths across various forms of adversity, such as stress (Harzer and Ruch, 2015), war and terrorism (Shoshani and Slone, 2016), natural disaster (Duan and Guo, 2015), at-risk/vulnerable populations (Duan and Wang, 2018), traumatic brain injury (Andrewes et al., 2014), suicidal inpatients (Huffman et al., 2014), psychopathology (Freidlin et al., 2017), addictions (Logan et al., 2010), aggression (Park and Peterson, 2008), and intellectual/developmental disability (Niemiec et al., 2017). Several of these studies support and discuss character strengths resilience; one study in particular found character strengths predict resilience over different positive phenomena such as self-efficacy, self-esteem, positive affect, social support, optimism, and life satisfaction (Martínez-Martí and Ruch, 2016). Niemiec (2020) documents studies linking each of the 24 character strengths with resilience.

### When Possible in Practice, Default on the Science

This approach involves having and integrating a solid grounding in character strengths science when introducing character strengths to a client. This foundation extends to practitioners favoring a mindset that they first turn to the scientific findings on character strengths when offering an intervention. In many instances, we have observed well-intentioned practitioners make something up and then link it back to “positive psychology research” explaining the activity as “based on evidence.” In this emerging scientific field, we suggest a more conservative approach: start with the science and then allow the practice to unfold from there. For example, start with intervention studies that have found using signature strengths to be superior to controls; use that as the practical strategy. If that is not an optimal avenue for your client, you might then turn to theoretical articles, correlation studies, or one activity within an evidence-based program. To flesh out this approach, Niemiec (2018) offered seven, non-sequential categories to guide practitioners in applying strengths, based on evidence; these were later discussed in Ruch et al. (2020) as pathways to justify a strengths-based intervention. A summary of these can be found in Table 3.

**Table 3:** Research-based framework to guide practitioners in applying character strengths.

Category	Reference Base (example)	Name from Niemiec (2018)	Description
Intervention from a controlled strengths intervention study	Harzer and Ruch (2016)	Strengths Alignment	List five work tasks, list five signature strengths, align at least one strength that could be used while working on any and all work tasks.
Variation of a controlled strengths intervention study	Gander et al. (2013)	Holistic Strengths Use	Building off the evidence around “use a signature strength in a new way,” this intervention involves exploration of a signature strength as expressed from the heart, the head/mind, intrapersonally, and interpersonally.
Controlled intervention study, with character strengths added in afterward to enhance effects	Loveday et al. (2018)	Best Possible Self with Strengths	Begins with the instructions of imagining a time in the future in which one is expressing one’s best self. The second step is to imagine the character strengths pathways one will need to express in order to make that best possible self a reality.
Intervention discussed in peer-reviewed works	Veldorale-Brogan et al. (2010)	Turn Your Strengths Other-Oriented	Direct one signature strength outward in a relationship to bring benefit to that person.
Intervention extrapolated from an observational study	Kashdan et al. (2018)	Character Strengths Appreciation	List three of one’s partner’s character strengths, an example for each, and convey appreciation to them – why they are valued for their strengths use.
Intervention extrapolated from a theoretical concept	Rempel et al. (2007)	Character Strengths Genogram	As one creates a standard family genogram, add three character strengths that describe each entry; look for patterns and discuss with family members.
Intervention within a multi-activity, research-supported program	Niemiec (2014); Pang and Ruch (2019a)	From Mindless to Mindful	Part of the evidence-based mindfulness-based strengths practice (MBSP) program, this involves choosing a bad habit/vice and each day examining the autopilot mind while engaging in the habit; then bring mindful attention and character strengths into action.

### Overuse, Underuse, and Optimal Use of Character Strengths

An exciting area for practitioners is examining character strengths overuse and underuse. New empirical work using the Overuse, Underuse, and Optimal-Use of Character Strengths Survey (Freidlin et al., 2017) has begun to discover overuse/underuse patterns related to diagnostic conditions, such as for social anxiety disorder (Freidlin et al., 2017) and obsessive-compulsive disorder (Littman-Ovadia and Freidlin, 2019). Central arguments, theory, concepts, research, practical strategies, and language for overuse and underuse have been articulated (Niemiec, 2019a).

Practitioners help clients identify the character strengths that are out of balance in challenging situations and relationship conflicts and discuss client strategies for finding balance – or to arrive at the golden mean for a particular situation – the right combination of strengths, expressed with the right intensity, and in the right situation. That said, there are currently no intervention studies that have tested the overuse of character strengths, which indicates that this intriguing dynamic has much to be explored.

### The Integration of Mindfulness and Character Strengths

The integration of these popular areas is of significant interest to practitioners. The weaving of character strengths to improve meditation and mindful living practices is referred to as “strong mindfulness” (Niemiec et al., 2012) while the using of mindfulness and mindful living to bring balance, savvy, and enhancement to character strengths is referred to as “mindful strengths use” (Niemiec, 2012). The 8-week program that guides participants through the boosting and integration of each is called mindfulness-based strengths practice (MBSP; Niemiec, 2014). Several theoretical, applied, and intervention studies offer a good evidence-base for MBSP. Intervention studies have shown benefits for well-being, engagement, meaning, health, and student retention (Wingert et al., 2020). Additional studies have found MBSP to be superior to the most widespread mindfulness program [mindfulness-based stress reduction (MBSR)] for boosting work task performance, workplace satisfaction, and the strength of humor (Hofmann et al., 2019; Pang and Ruch, 2019a).

In the practitioner survey, the integration of mindfulness and character strengths was more common in personal practice than in application with clients.

### Use the Character Strengths Model: Aware, Explore, Apply

The most straightforward character strengths process is the three-phase model, Aware-Explore-Apply (Niemiec, 2014) which entails: first, raising awareness of a character strength the client was previously unaware of or had limited use of; next, co-exploring the character strength with questions, activities, reflections, and challenges; and finally, moving into the application as the client chooses concrete goals and next steps for putting the character strength into action. These phases have been studied and revealed positive results, including a boost to thrive and decrease in negative emotions (Bu and Duan, 2018) and increases in strengths use and well-being (Dubreuil et al., 2016). This model can be applied in any field in which working on character strengths is part of the focus.

### Keep a Personal Character Strengths Practice

As with teaching other practices, it’s important the practitioner first applies the practice to themselves (e.g., for mindfulness, see Dunn et al., 2012). This facilitates the “know thyself” and “practice what you preach” adages common in areas of self-development, and it enhances the understanding, depth, and facility when later working with a client’s character strengths. There are many ways to set up a practice with character strengths (which can, in turn, be taught to clients). Four main practice pathways from Niemiec (2018) include:

- *Formal*: having a regular practice with strengths, often the same time each day or week, e.g., practicing gratitude every evening by counting three good things that happened at the end of each day; or having a strengths appreciation conversation with one’s relationship partner every Sunday morning.
- *Informal*: using character strengths when needed such as at times of stress, e.g., when one’s body feels tight from stress, one pauses to breathe and consider which of their character strengths they could immediately bring forward.
- *In-the-moment*: looking to daily routines and areas of life taken for granted for character strengths to be discovered, e.g., while reflecting/journaling, a person realizes they have already been using their appreciation of beauty, prudence, and curiosity as they take their dog for a walk.
- *Cued*: use of the external environment to cue or remind the individual to use their character strengths, e.g., the individual arranges that every time they hear a bell in their environment, they will pause and consider how they can use one of their signature strengths.

### Target Specific Strengths

A number of strength practitioners focus on one particular character strength in their practice with clients (37%). There is an extensive literature on each of the 24 strengths (Peterson and Seligman, 2004) so focusing on a specific character strength can have a scientific foundation. The practitioner should be familiar with intervention studies supporting the targeted strength, such as for the strength of hope, being familiar with interventions such as teaching clients about agency and pathways thinking (Snyder, 2000). This is an emerging approach that offers practitioners a simple inroad into helping clients, although it’s important to point out it can be narrow and limiting if one or two-character strengths are the sole focus or the only tools in the practitioner’s armamentarium.

## Ripe with Potential Practices

These are areas that are strong conceptually yet empirical research is scant. In workshops and trainings for practitioners, these are usually received with significant enthusiasm and curiosity. Several of these areas reflect character strengths dynamics. This is not an exhaustive list and is meant to offer initial ideas for researchers to investigate and for practitioners to work with and offer observations to researchers. Further exploration and examples for each can be found in Niemiec (2018).

### Phasic Strengths

These are strengths of an individual that are not signature strengths, yet the individual brings forth the strength strongly when the situation calls for it (Peterson and Seligman, 2004). A person who’s not high in zest might bring forth significant energy and enthusiasm when presenting to students. Despite being discussed in the original text of the *VIA* Classification, including a tentative measurement tool called the Rise to the Occasion Inventory (Peterson and Seligman, 2004), we are not aware of any empirical studies assessing or examining phasic strengths. Some observations have been made about these strengths as situational strengths (Escandón et al., 2016), and some conceptualizing has been done on phasic strengths and stress (Niemiec, 2019b). This is where the practice runs ahead of the research as practitioners ask clients about phasic strengths and explore situations in which clients rise to the occasion with character strengths at uncertain and challenging times.

### Hot-Buttons

Hot buttons are sensitive areas in which another person’s perceived strengths overuse or underuse triggers discomfort/frustration in the observer. This might stem from the observer’s own character strength beliefs, preferences, or expectations. Hypothetically, the observer’s character strength has been affronted or offended in a way that feels personal and deliberate. This area is ripe for research investigation and for practitioners to explore relational conflicts and troubling interactions clients have.

### Receiving Character Strengths

Most of the research and practice on character strengths has focused on inwardly and outwardly expressing one’s character strengths. What about how the character strength is received by the other? First introduced as a character strength name, the “capacity to love and be loved,” Peterson and Seligman (2004) may not have realized they were touching upon an interesting strength dynamic. Pileggi Pawelski and Pawelski (2018) advanced this dynamic by highlighting how gratitude is given and received in couples. We argue that all 24 character strengths have this characteristic, however, research on the topic is sparse. Observationally, how a relationship partner receives humor from their partner’s frequent use of humor might dictate whether the relationship will deepen or be constrained. The expression of forgiveness by someone can be herculean in terms of the emotional toll and therefore how the forgiveness is received by the other can be an important factor in the giver’s healing.

### Character Strengths Collisions

A character strengths collision can occur intrapersonally or interpersonally and refers to the dynamic when two character strengths are opposed to one another and are eliciting an internal or external tension/conflict.

### Character Strengths Synergies

These are win-win situations in which the character strengths of two or more people combine and are greater than the sum of the parts. Synergies can also occur internally with character strengths expressed together to a positive effect.

### The Tempering Effect and Towing Effect

Described in the context of overuse and underuse of character strengths in Niemiec (2019a), these dynamics occur when one character strength is used to bring balance to another character strength. The tempering effect refers to the use of character strength to help manage a higher strength, for example, using self-regulation to temper one’s curious questioning. The towing effect refers to the use of a higher character strength (e.g., signature strength) to boost or tow-along a lower character strength, for example, the use of one’s top strength of the love of learning to read about and explore new knowledge about how to use one’s lower strength of humility.

## The Research on Character Strengths: Soaring, Emerging, or Ripe with Potential?

We use the same framework – soaring, emerging, ripe with potential – for the current status of the research on character strengths. The first author has been tracking the science of character for more than a decade and an exhaustive summarized list of over 700 studies can be found categorized on the *VIA* Institute website (VIA Institute, 2021). Note that this number does not include the thousands of studies that have amassed on particular character strengths (e.g., creativity, hope, leadership, love), rather it represents studies using a *VIA* Survey assessment measure (there are 17 validated measures available to any researcher), the *VIA* Classification, or clusters of specific character strengths (e.g., studies of the character strengths under the transcendence virtue, Huta and Hawley, 2010).

As opposed to an exhaustive list of research areas or domains that are soaring, emerging, or ripe with potential, we selected a handful of examples of domain areas for each of the three categories. These examples are offered to catalyze researchers to build off of what is soaring or emerging or to consider pursuing areas that would benefit from growth.

### Soaring Research Domains

To be an area of research that is soaring, we considered domain areas that have at least 25 studies that explored the science of character in that domain. The domains of work/organizations and education meet this criterion (see VIA Institute, 2021). While still neophyte character strength domains, these areas have examined situations within their respective domain, replicated findings, offered basic and applied research, and deployed a number of character strengths concepts for further research and practice. While we frame these as “soaring,” we want to highlight the observation that there is far more that we do not know about the application of character strengths in work and education than we do know. That said, a strong foundation is being built for not only researchers but also practitioners to explore and advance.

The workplace has been the most thriving domain in the study of character strengths as character strengths relate to a number of positive and ambitious workplace behaviors (Gander et al., 2012). A range of strengths-related outcomes include job performance (Harzer and Ruch, 2014), job satisfaction, work engagement, and work well-being (Miglianico et al., 2019), improved workplace climate (van Woerkom and Meyers, 2014), employee levels of self-efficacy and proactive behavior (van Woerkom et al., 2016), and improved coping with stress at work (Harzer and Ruch, 2015), to name a few. The importance to both managers and employees of character strengths awareness, alignment with work tasks, and appreciation among colleagues is substantial (Mayerson, 2015).

Novel findings with employees’ top strengths have been conducted and found that signature strengths are connected with positive work experiences, irrespective of which character strengths of the 24 are highest (Harzer and Ruch, 2013). Another study found that workers who used four or more of their signature strengths at work had more positive work experiences and work-as-a-calling than those who used less than four signature strengths (Harzer and Ruch, 2012). A study with work supervisors support found that employees who received supervisor support around character strengths (but not colleague support) increased their character strengths use the following day (Lavy et al., 2016). Different subset categories of character strengths (e.g., lower strengths, happiness strengths) have been examined in the workplace with interesting results. For example, Littman-Ovadia et al. (2016) found that the subsets of signature, lower, and happiness strengths were each associated with positive outcomes, but for work performance, organizational citizenship behavior, and (less) counterproductive work behavior, signature strengths contributed most while for work meaning, engagement, and satisfaction, the happiness strengths contributed most.

The second soaring domain in the science of character strengths is education. Positive education examines character strengths patterns and interventions in children and adolescents within and outside of the school context. Character strengths have been articulated as central to the educational experience of young people and a number of practices for the classroom setting have been discussed (Linkins et al., 2015; Darwish and Niemiec, 2021). Character strengths have been outlined as central for boosting 21st-century competencies relating to cognitive, interpersonal, and intrapersonal competencies as identified by the American National Research Council (Lavy, 2019). In addition, systems thinking and systems-wide implementation of character strengths are crucial for this domain (Darwish and Niemiec, 2021).

A wide range of positive classroom outcomes have been found such as positive affect, negative affect, and school achievement (Weber et al., 2016), well-being (Oppenheimer et al., 2014), strengths use, class cohesion, relatedness, and less class friction (Quinlan et al., 2014), as well as social relationships, school performance, and academic motivation (Grinhauz and Castro Solano, 2014).

Intervention studies of programs from different parts of the world have shown positive findings. In the United Kingdom, a study evaluated the impact of a character strengths program on adolescents and found that adolescents who participated in the character strengths exercises had significantly higher life satisfaction than adolescents who did not participate (Proctor et al., 2011). In a Chinese educational context, a strengths training intervention was found to be effective in boosting life satisfaction in the short‐ and long-run (Duan et al., 2013). Some positive education programs which have character strengths as core to the program have found increases in academic scores, social skills, and students’ enjoyment and engagement in school, as well as improve character strengths such as curiosity, love of learning, and creativity (Seligman et al., 2009). In New Zealand, a strengths-spotting intervention of teachers found benefits for improving student outcomes which were explained by better classroom engagement, positive affect, and needs satisfaction (Quinlan et al., 2019). In India, randomized controlled trials involving thousands of girls in poverty found that those who received a curriculum which incorporated character strengths (i.e., identification and use of signature strengths and concrete examples of using other strengths) exhibited significantly greater physical health and psychosocial health benefits in comparison to those girls who received a similar curriculum which did not include character strengths and girls who did not receive any curriculum at all (controls; Leventhal et al., 2016). In Australia, while not an intervention study, the integration of character strengths knowledge and activities into an entire school revealed a number of benefits for teachers and students and is documented in White and Waters (2014).

### Emerging Research Domains

For the category of emerging domains, we identified domains with at least 10 peer-reviewed/scholarly articles on character strengths in the domain and were published recently (within the last 5 years) indicating a spike of interest. This points to a new literature beginning to emerge, perhaps reflecting enthusiasm from research groups and scholars claiming an interest in the area. We discuss two domains: health/medicine and mindfulness.

Character strengths have been examined across various dimensions of physical health, including healthy eating, physical fitness, personal hygiene, substance avoidance, and living an active way of life, finding some character strengths more relevant in each area (Proyer et al., 2013a). A randomized controlled trial with seriously ill children found that a “granting a wish” intervention reduced nausea and increased life satisfaction, positive emotions, and strengths, compared to a control group (Chaves et al., 2016). Niemiec and Yarova (2019) reviewed the implication of character strengths integration for health across three levels – the individual, the healthcare provider, and the system.

Intervention studies have brought character strengths in as one piece of a healthcare program and received positive feedback from patients as some of the most impactful elements. For example, patients suffering from acute coronary syndrome benefitted from an 8-week phone intervention which included identification and use of a signature strength (Huffman et al., 2016). A number of significant findings surround the integration of character strengths with physicians (Strecker et al., 2019), including the connections with physician work engagement and well-being (Huber et al., 2019), and the mutual impact of signature strengths applications and perceived hospital climate (Höge et al., 2019).

The integration of mindfulness and character strengths was mentioned earlier as an emerging practice. The research has received similar support with ~20 publications since the development of the first positive psychology program to integrate mindfulness with positive qualities in a systematic way – MBSP (Niemiec, 2014). MBSP has received theoretical support for its two-way, mutual integration (Pang and Ruch, 2019b) and there are several intervention studies with positive findings (e.g., Wingert et al., 2020). A wide range of application areas have been explored with MBSP (e.g., Bretherton and Niemiec, 2020), for example, supervision (Sharp and Rhinehart, 2018), early childhood development (Lottman et al., 2017), meaning in life (Littman-Ovadia and Niemiec, 2017), and intellectual/developmental disability (Shogren et al., 2017).

Additional areas that meet or nearly meet the criteria for emerging research domains with character strengths include military, positive psychotherapy, positive parenting, intellectual/developmental disability, workplace/team roles, overuse/underuse/optimal-use, stress management, and positive relationships.

### Ripe With Potential Research

For the ripe with potential domain, we selected areas in the science of character strengths that have between zero and three studies and the potential contribution of character strengths is robust and synergistic. We highlight three areas that are ripe for character strengths integration: spirituality, environment/nature connection, and peace/conflict studies. Each has seedlings emerging yet is wide open for extensive scientific investigation and eventually best practices.

The integration of spirituality and character strengths has been piecemeal with spirituality links to particular character strengths such as forgiveness, gratitude, humility, and love. The mutual synergy informed by the latest character strengths concepts, hundreds of studies in character science, character strengths interventions, and new research in spirituality has been largely unexplored. Niemiec et al. (2020) approached these areas by laying out a map of the six existing levels of integration for spirituality within the *VIA* Classification, and offered models for exploring this integration in the context of the psycho-spiritual journey toward wholeness. They offer two theoretical pathways by which character strengths and spirituality integrate and mutually benefit one another – the grounding path (where strengths offer tangibility and thereby deepen spirituality) and the sanctification path (where spirituality can elevate character strengths) and expound on several integration practices for each pathway that are grounded in science. Another article (Littman-Ovadia and David, 2020, this issue) shares how character strengths contribute to non-dual spirituality. Future studies might examine these pathways of integration and the practices therein.

The area of environment/nature connection also represents significant potential for the importance of character strengths. Considering the wide-ranging benefits of character strengths applications, it would seem reasonable to believe there would be a contribution to both pro-environmental behaviors and nature connectedness. One study showed character strengths were connected with sustainable behaviors, defined as actions intended to protect the socio-physical resources of the planet (Corral-Verdugo et al., 2015). Another study examined psychological barriers to environmental self-efficacy and found certain character strengths were strongly related (e.g., zest and leadership) and others were related but less strongly (e.g., kindness, humility, prudence, fairness, and forgiveness; Moeller and Stahlmann, 2019). Work on the integration of MBSP and nature connectedness/pro-environmental behaviors is in the beginning stages.

Peace studies (or peace/conflict studies) is the area that surprises us most that there has not been extensive research integrating character strengths to date. Cohrs et al. (2013) offered ways in which positive psychology contributes to peace and point out that character strengths offer strategies for inner peace and peace of mind and might contribute to peace, nonviolence, reduced reactivity, and building a global resilience.

In the literature on peace, a common distinction is made between positive peace and negative peace, where positive peace refers to the creation or building up of harmony and equity while negative peace refers to the decrease or elimination of violence, war, and human conflict (Christie et al., 2008; Neto and Marujo, 2017). In addition, there are many types of peace including inner/personal peace, relational peace, intragroup peace, intergroup peace, and international peace. Character strengths would seemingly have a significant place in positive and negative peace across each of these levels. The first author has begun an investigation of the role of character strengths with these levels.

Additional areas we believe are ripe with potential include social/racial justice, positive leadership, addictions and psychopathology, and sport/performance psychology.

### Conclusion

The science of well-being, or science of positive psychology, was conceived as a bridge between academic scholarship, practical wisdom, and applied psychology/self-development. It is enveloped with many scholars, researchers, and practitioners eager to advance the field. One of the challenges is the siloed nature of the work. One of our aims with this paper has been to catalyze dialogue for scientists and practitioners by offering definitions, principles, and trending areas to unify disparate scientists and practitioners and spur collaborations.

We suggest the need for more seminal thought leadership papers and basic research in the areas mentioned as ripe with potential, and for researchers to take the next steps in examining the areas in the soaring and emerging domains. From a big-picture vantage point, the work in all these areas is only beginning; there are many nuances and challenges to untangle and discover in advancing the science of character strengths (Ruch et al., 2020).

We encourage practitioners to deepen their study of the principles of character strengths outlined and consistently engage in research-based practices with character strengths, which includes using the science as the default, having your own personal practice with character strengths, and taking action with practices such as strengths-spotting, signature strengths exploration, integration with mindfulness, and adhering to character strengths models such as aware-explore-apply.

We have found – and as noted here the science supports this – character strengths play a substantial role for both the boosting of well-being and the handling of adversity. Each is mountainous areas for researchers and trained practitioners to continue exploring in the pursuit of understanding and benefiting the human condition.

## Data Availability Statement

The raw data supporting the conclusions of this article will be made available by the authors, without undue reservation.

## Ethics Statement

Ethical review and approval was not required for the study on human participants in accordance with the local legislation and institutional requirements. Written informed consent for participation was not required for this study in accordance with the national legislation and the institutional requirements.

## Author Contributions

RN researched, drafted, and revised the paper. RP lead the practitioner survey discussed and revised the paper. All authors contributed to the article and approved the submitted version.

### Conflict of Interest

The authors declare that the research was conducted in the absence of any commercial or financial relationships that could be construed as a potential conflict of interest.
